# Self-assembly of hierarchical MoS_x_/CNT nanocomposites (2<x<3): towards high performance anode materials for lithium ion batteries

**DOI:** 10.1038/srep02169

**Published:** 2013-07-09

**Authors:** Yumeng Shi, Ye Wang, Jen It Wong, Alex Yuan Sheng Tan, Chang-Lung Hsu, Lain-Jong Li, Yi-Chun Lu, Hui Ying Yang

**Affiliations:** 1 Pillar of Engineering Product Development, Singapore University of Technology and Design, Singapore 138682, Singapore; 2 Institute of Atomic and Molecular Sciences Academia Sinica, Taipei 10617, Taiwan; 3 Department of Materials Science & Engineering, National Chiao Tung University, HsinChu 300, Taiwan; 4 Department of Physics Natonal Tsing Hua University, HsinChu 300, Taiwan; 5 Department of Mechanical and Automation Engineering, The Chinese University of Hong Kong, Hong Kong SAR, China

## Abstract

Two dimension (2D) layered molybdenum disulfide (MoS_2_) has emerged as a promising candidate for the anode material in lithium ion batteries (LIBs). Herein, 2D MoS_x_ (2 ≤ x ≤ 3) nanosheet-coated 1D multiwall carbon nanotubes (MWNTs) nanocomposites with hierarchical architecture were synthesized via a high-throughput solvent thermal method under low temperature at 200°C. The unique hierarchical nanostructures with MWNTs backbone and nanosheets of MoS_x_ have significantly promoted the electrode performance in LIBs. Every single MoS_x_ nanosheet interconnect to MWNTs centers with maximized exposed electrochemical active sites, which significantly enhance ion diffusion efficiency and accommodate volume expansion during the electrochemical reaction. A remarkably high specific capacity (i.e., > 1000 mAh/g) was achieved at the current density of 50 mA g^−1^, which is much higher than theoretical numbers for either MWNTs or MoS_2 _along (~372 and ~670 mAh/g, respectively). We anticipate 2D nanosheets/1D MWNTs nanocomposites will be promising materials in new generation practical LIBs.

Advanced energy storage technology is the key to manage the energy supply and demand. Lithium ion batteries (LIBs) have attracted increasing research interests and become one of the main power sources for portable electronic devices and electric vehicles due to its high energy densities, no memory effect, and good cycling stability compared to other alternatives^1^. In commercial LIBs, graphite and lithium metal oxides are commonly employed as the negative (anode) and positive (cathode) electrode materials, respectively. Lithium is the lightest metal that delivers high energy density per electron with a theoretical electrochemical capacity of Li to Li^+^ is 3860 mAh/g^2^. However, further advancements in the state-of-the art LIBs are still bottlenecked by the limitation in the anode materials associated with limited capacity (i.e., graphite, ~372 mAh/g), lack of shape flexibility and low ion/electron conductivity^3^,^4^. In the past few years, substantial research efforts have been devoted in developing high performance LIBs electrodes. Various carbon nanomaterials, such as one dimension (1D) carbon nanotubes (CNTs)^5^,^6^, two dimension (2D) graphene nanosheets^7^,^8^, three dimension (3D) graphene foam^9^,^10^, have all been investigated as the anode materials in reversible storage of Li^+^, due to their outstanding electronic conductivities, high charge mobilities and large specific surface areas. As one of the crystalline form of carbon, 1D CNTs has high electric conductivity, good mechanical property, chemical stability and reversible redox reaction capability, which makes it a promising candidate as lithium insertion hosts for LIBs.

The nanostructured multifunctional heterostrucutres have been proved to work synergistically with both high capacity and good cyclability^11^,^12^,^13^,^14^. Molybdenum disulfide (MoS_2_), an inorganic graphite analogue, belongs to the layered transition-metal dichalcogenide (LTMDs) family. The weak van der Waals interaction between MoS_2_ layers allows the Li^+^ ions to diffuse without a significant increase in volume expansion and prevent the pulverization problem of active materials caused by the repeatly lithiation and delithiation process. The promising potential of MoS_2_ serving as an anode materials for LIBs is widely reported in the literature due to its attractive specific capacity^15^,^16^,^17^,^18^,^19^,^20^,^21^. Theoretically the conversion reaction between Li ions and MoS_2_ leads to four moles of lithium incorporation per mole of MoS_2_ accounting for 670 mA h g^–1^ lithium storage capacity that is ~1.8 times higher than the graphite electrode^20^. With all these significant advantages, MoS_2_ has attracted lots of research interests and became a promising material as an anode material in LIBs^17^,^18^,^19^. Various methods have been reported for the synthesis of MoS_2_ including the gas-phase reaction of MoO_3_ with H_2_S or S vapor^22^,^23^, thermal decomposition of ammonium thiomolybdate^24^,^25^, and solvent thermal method^26^,^27^.

The solvent thermal process is an important wet chemistry synthesis method and has been widely used to prepare various nanomaterials or nanocomposites. It has been reported CNTs favored the growth of the tubular MoS_2_ on the surface of carbon nanotube side walls and promoted the formation of tubular MoS_2_ layers with high crystallinity^27^,^28^,^29^, CNTs/MoS_2_ composites have also been prepared by the simple solvothermal method^30^,^31^. For example, tubular MoS_2_ layers coating on CNTs were synthesized by the hydrothermal reaction between Na_2_MoO_4_ and CS(NH_2_)_2_ with the presence of CNTs^12^. The surface area of MoS_2_ is limited by the surface area of CNTs. Nevertheless, when aqueous solvent is used, CNTs need to be treated by refluxing in high concentrated strong acid in order to improve the wetting between CNTs and MoS_2_ precursor^28^. This acidic treatment will introduce defects in CNTs and negatively affect the electrical properties of CNTs. MoS_2_/CNTs with a design of 2D MoS_2_ nanoflakes surrounded by a coating of CNTs was synthesized by using Na_2_MoO_4_ and KSCN as reactant and ethylene glycol as solvent in the presence of CNTs^27^. These composites show higher capacity and improved cycling stability compared to pure MoS_2_. The MoS_2_ nanoflakes synthesized are relatively thick and randomly attached to CNTs, which causes a continues capacity fading during cycles^27^. Wang *et al.* prepared MoS_2_ overlayers supported on coaxial CNTs by wet-chemistry process and studied the reversible lithium-storage behaviors of this composite^32^. A reversible capacity of 400 mAh/g was achieved; however this value is much smaller than the non-coaxial MoS_2_/CNTs composite.

## Results

**Figure 1:**
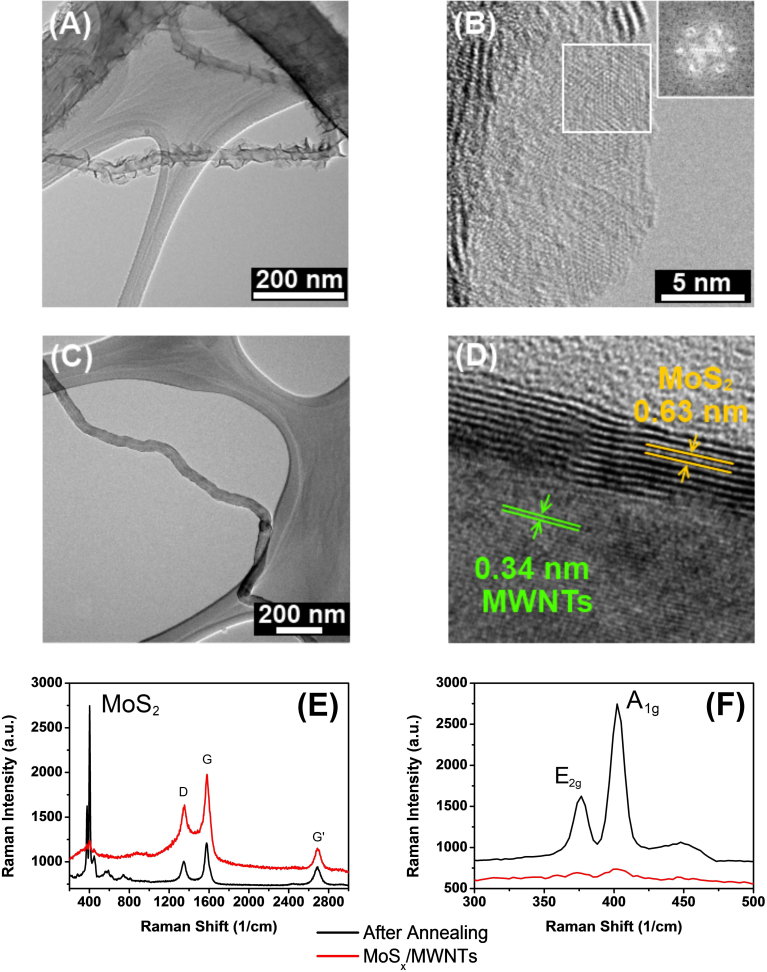
(A) and (C) Low-magnification TEM image of MoS_x_/MWNTs with hierarchical nanostructure and MoS_x_/MWNTs after annealing at 800°C under Ar protection, (B) and (D) HRTEM images of a free standing monolayer MoS_x _and the side wall of the composite after annealing. Inset in Figure 1 (B) shows the FFT pattern taken from the marked area. (E) Raman spectra of the MoS_x_/MWNTs. Figure 1 (F) compares the magnified Raman signature of ta prepared MoS_x_/MWTNs and the one after annealing.

Herein, we report a unique MoS_x_/CNTs (2 ≤ x ≤ 3) nanostructure synthesized by simple solvent thermal method at low temperature (200°C) using (NH4)_2_MoS_4_ as single reactant and N,N-dimethylformamide (DMF) as solvent in the presence of MWNTs. The synthesized MoS_x_/MWNTs composites are different from the previous report for MoS_2 _sheath/CNT-core nanoarchitecture^32^, the MoS_x_ layers are not confined to the MWNTs surface, but extend the layered structure out of the cylindrical tubules (as shown in Figure S1). To understand the forming of hierarchical architecture, the morphology and lattice structure of as prepared MoS_x_/MWTNs composite was compared with the samples treated under elevated temperature. Figure 1 (A), (B) show the TEM images of MoS_x_ coated MWNTs prepared by the solvent thermal method. The HRTEM in Figure 1 (B), gives a close-up view of the MoS_x_ branch attached on MWNTs surface. The inset shows a fast Fourier transform (FFT) pattern taken from the marked area in Figure 1 (B). The HRTEM and FFT results indicate the semi-crystalline nature of the MoS_x_ layers. As seen in Figure 1 (C) and (D) MoS_2 _sheath/CNT-core nanoarchitecture was obtained by thermal annealing at 800°C under Ar protecting environment. The two layered spacing can be identified to be around ~0.62 and ~0.34 nm, which are in good consistence with the value for MoS_2_ layers and the lattice spacing between the graphitic planes of MWNTs. Figure 1 (E) and (F) compare the Raman spectra taken from the as obtained MoS_x_/MWNTs samples and thermal treated MoS_2_ sheath/CNT-core nanocomposites. The Raman Peaks at around 1347 and 1576 cm^−1^ belong to MWNTs. The G′ band of MWNTs locates at 2686 cm^−1^. The Raman Peaks of MoS_2_ appear at 376 and 402 cm^−1^. It was also found that the Raman signature of MoS_2_ dramatically increased after thermal annealing, which suggests the formation of highly crystallized MoS_2_ layers. This is agreed with the result of HRTEM.

**Figure 2:**
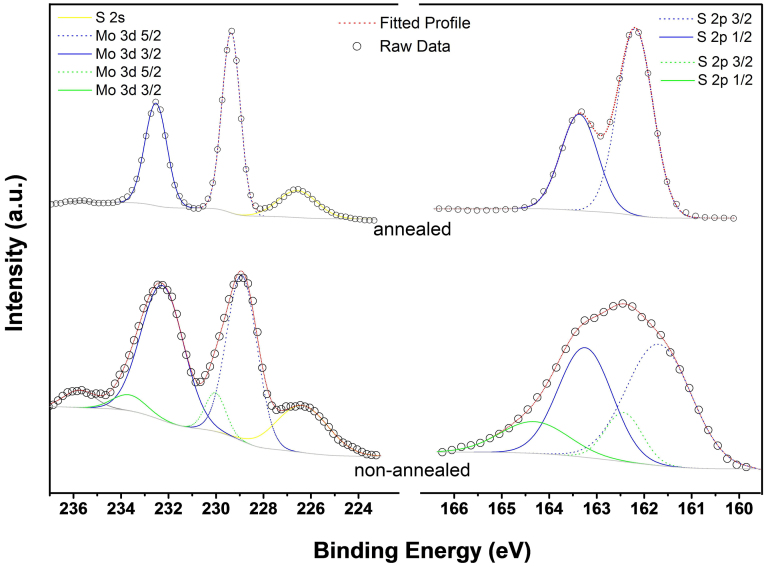
Chemical composition analysis by X-Ray photoemission spectroscopy (XPS) for Mo and S. The lower and upper cures display the corresponding spectrum taken from the as obtained and 800°C annealed MoS_x_/MWNTs samples respectively.

X-ray photoelectron spectroscopy (XPS) was used to investigate the chemical states of Mo and S in the MoS_x_/MWNTs nanocomposites. Figure 2 displays the XPS characterization of the samples before and after thermal annealing at 800°C under Ar protecting environment. The high-resolved XPS spectra shows the binding energies of Mo 3d 3/2, Mo 3d 5/2, S 2p ½ and S 2p 3/2 peaks in the thermal annealed MoS_x_/MWNTs are located at 232.4, 229.2, 163.3 and 162.1 eV, respectively, indicating that Mo^4+^ existed in the annealed MoS_x_/MWNTs^32^. The stoichiometric ratio of S:Mo estimated from the respective integrated peak area of XPS spectra is ~2.125 suggesting the structure is close to MoS_2_. For the as prepared MoS_x_/MWNTs two broaden peaks centered at ~232.5 and ~228.9 eV, in addition to the XPS peaks for MoS_2_ structure, other sets of peaks are also observed. The higher energy shift of Mo 3d_3/2_ and 3d_5/2_ doublet are associated with higher valence states. The observation of Mo 3d_3/2_ and Mo 3d_5/2_ peaks at 233.6 and 230.5 eV with separation energies close to 3.1 eV can be attributed to the presence of Mo^5+^ ions^33^,^34^. For the non-annealed MoS_x_/MWNTs the S 2p spectra can be interpreted in terms of two doublets, with S 2p3/2 binding energies of 161.7 and 163.2 eV. Compared to the thermal annealed samples, the additional S 2p1/2 and 2p3/2 energies located at 164.3 and 163.2 eV can be assigned to the binding energies of apical S^2−^ or bridging disulfide S_2_^2−^ ligands. The S 2p spectrum that can be fit with two S 2p doublets, which is similar to those of amorphous MoS_3_^35^,^36^. The presence of bridging apical S^2−^ or bridging S_2_^2−^ is in good consistence with the TEM analyses in Figure 1 (B), which reveals that the MoS_x_ obtained are basically semicrystalline. Furthermore, the S/Mo elemental ratio estimated from the integrated peak area of XPS spectra is ~3.0 which also suggests the as grown MoS_x_ is stoichiometrically close to MoS_3_. The thermal decomposition of (NH4)_2_MoS_4_ is accompanied by molybdenum-sulfur redox processes, which include the oxidation of S^2−^ ligands of the MoS_4_^2−^ anion and the reduction of Molybdenum metal from Mo^VI^ to Mo^IV^, and various thermal decomposition intermediate may exist^37^. The XPS results confirm the presents of MoS_3_ while the Raman spectra from the as prepared samples show smaller but visible Raman Peaks of MoS_2 _at 376 and 402 cm^−1^ (as shown in Figure 1 (E)). Therefore, the exact phase of the MoS_x_/MWNTs compound is suggested to be a mixture of MoS_2_ and MoS_3._

**Figure 3:**
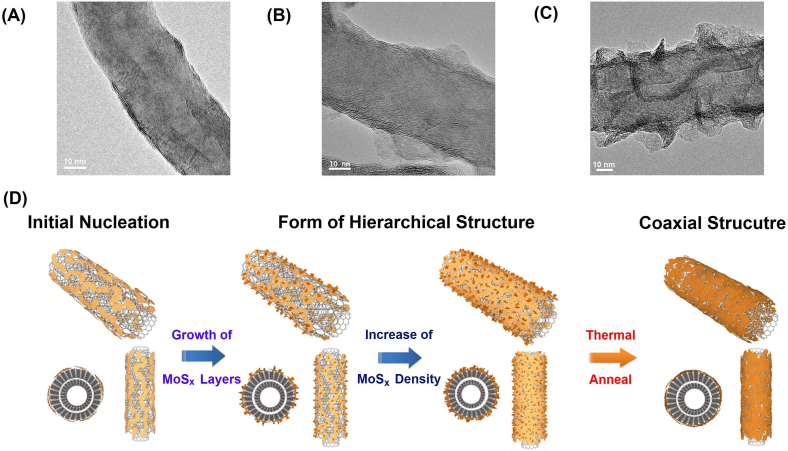
(A),(B) and (C) Low-magnification TEM images of MoS_x_/MWNTs with synthesized with increasing MoS_x_/MWNTs ratio (1:40, 1:20, 1:10), (D) shows the proposed growth mechanism for forming MoS_x_/MWNTs hierarchical structure.

The growth mechanism of MoS_x_/MWNTs layered structures were also investigated by varying the Mo/Carbon ratio in the precursor. Figure 3 (A), (B) and (C) show the TEM images of typical MoS_x_/MWNTs composites prepared with Mo/Carbon ratio of 1:40, 1:20 and 1:10. Figure 3 (D) proposes the growth mechanism of the MoS_x_/MWNTs composites. With limited amount of MoS_x_ precursor the MoS_x_ forms small segments on the sidewall of MWNTs. The hierarchical structure of MoS_x_ forms and the MoS_x_ layer structure extruding from the sidewall of MWNTs with the increase of Mo/C ratio. For the high concentration precursor, the MoS_x_ layers form uniformly on MWNTs. It has been reported CNTs favored the growth of the tubular MoS_2_ on the surface of carbon nanotube side walls and promoted the formation of tubular MoS_2_ layers with high crystallinity^27^,^28^, therefore at elevated temperate the MoS_x_ converted to MoS_2_ and form MoS_2 _sheath/CNT-core nanoarchitecture^32^.

## Discussion

Compared to the conventional MoS_2_/MWNTs structure, the novel MoS_x_/carbon composite has a three dimensional (3D) hierarchical structure, where the 1D multi wall carbon nanotube (MWNTs) as back bones, while the 2D MoS_x_ layers grown on the surface of MWNTs with a partially free standing branch like feature, which provide a large surface area of the active material to accommodate Li^+^. The hierarchical structure of MoS_x_/CNTs, could effectively combine the merits of the good electrical conductivity of CNTs and excellent electrochemical performance of individual MoS_x_ layer throughout cycling. Due to the excess of sulphur in MoS_x_ an increased layer distance of S–Mo–S can be expected, which results in less strain and smaller intercalation barrier of Li ions. Meanwhile, the CNTs used in this work have a long tube length, which creates large internal voids in the composites that could absorb and buffer the mechanical stress which caused by the local volume variation during lithium insertion and extraction.

**Figure 4:**
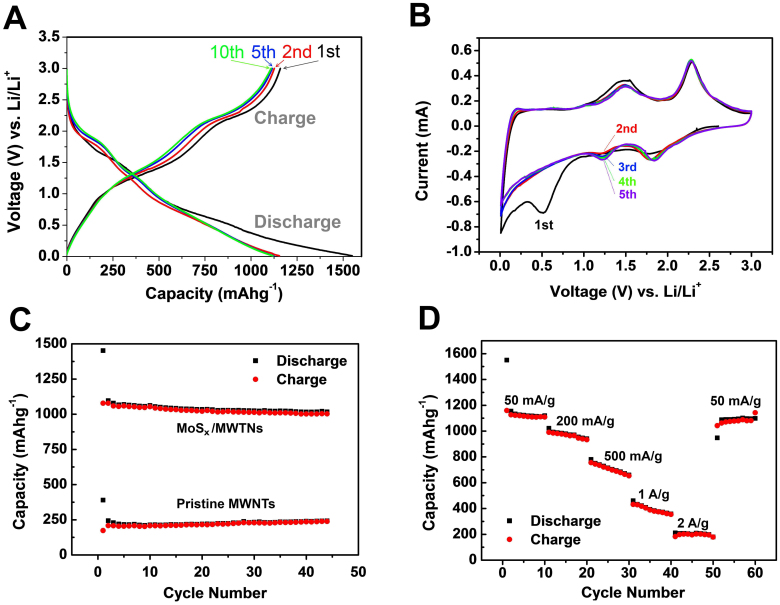
(A) Voltage profiles of MoS_x_/MWNTs charged-discharged at 50 mA g^−1^, (B) Representative cyclic voltammograms of MoS_x_/MWNTs composite for the first 5 cycles at a scan rate of 0.5 mVs^−1^ between 0.01 V and 3 V. (C) comparison of cycling stability between MoS_x_/MWNTs and MWNTs charged-discharged at 50 mA g^−1^), and (D) Rate capability of MoS_x_/MWNTs charged and discharged at various current densities.

Considering the electrodes with special hierarchical nanocomposites are advantageous to LIBs, we investigate the lithium storage properties of as-prepared MoS_x_/MWNTs using half-cell configuration. Figure 4 shows the electrochemical performance of MoS_x_/MWNTs as anode materials. Figure 4 (A) illustrates the first, second, fifth and tenth discharge/charge voltage profiles of the MoS_x_/MWNTs composite electrode in the voltage range of 0.01 to 3 V (vs. Li/Li^+^). During the first discharge, the initial discharge capacity between 2.0 to 1.5 V can be attributed in part to the reaction of residual carbon (MWNTs) surface functional group^38^ and in part to lithium insertion into the MoS_x_/MWNTs composites forming Li_n_MoS_x _(0 < n < 4)^39^, according to the reaction MoS_x_ + nLi^+^ + ne^−^ → Li_n_MoS_x_^27^,^40^. We note that it is previously proposed that a better formulation for MoS_3_ would be Mo^V^_2_(S_2_^2−^)(S^2−^)_4_, therefore, the reduction of sulfur during initial discharge can also be considered here^39^. Following this, the capacity between 1.0 to 0.5 V can be attributed to the conversion reaction process MoS_x_ + 2xLi^+^ + 2xe^−^ → Mo + xLi_2_S^41^,^42^,^43^. The metal sulfide reacts with lithium ions forming metal nanoparticles and insoluble Li_2_S matrix^20^. It was argued that the nanosized metal particles promote the reversible reaction which is responsible for the reversible lithium-storage capacity, therefore the phase segregation of transition metals should be limited in order to improve the cycling stability^32^. The sloping plateau at the lower voltage region (below 0.5 V) includes the contribution from the formation of a solid electrolyte interface (SEI) and the gel-like polymeric layer on the surface of the active materials^44^. In the subsequent charge process, a plateau at ~1.3 V and the sloping region above 2.2 V are attributed to the oxidation of Mo particles to MoS_x_ and the oxidation of Li_2_S to form S, respectively^42^,^45^,^46^. We note that lithium extraction from the Li_n_MoS_x_ phase should also be considered here^27^,^39^,^40^. The initial discharge and charge capacities are found to be 1549 and 1159 mAhg^−1^, respectively. (with a Coulombic efficiency of 74.8%).The irreversible capacity loss of approximately 25.1% in the 1^st^ cycle can be mainly attributed to the irreversible processes including the electrolyte decomposition and inevitable formation of the SEI, which have been observed for nanosized anode materials^47^. During the 2^nd^ cycle, the discharge capacity decreases to 1154 mAh/g with a corresponding charge capacity of 1126 mAh/g, leading to a much higher Coulombic efficiency of 97.5%. This value further increased to 99.6% in the 5^th^ cycle and still maintained above 98.6% at the 10^th^ cycle. To further clarify the electrochemical process of the MoS_x_/MWNTs composite, cyclic voltammograms (CV) measurement of the first three cycles in the voltage range of 3.0 – 0.01 V with a scan rate of 0.1 mVs^−1^ was shown in Figure 4 (B). In the first cycle a very small reduction peak at ~1.80 V was found, which can be related to the reaction of residual carbon surface functional group^38^, in part to lithium insertion into the MoS_x_ structure forming Li_n_MoS_x_^39^, and the reduction of traced sulfur^39^. A pronounced reduction peak at ~0.50 V was observed in the first cycle, however for the subsequent cycles, the peak at ~0.50 V disappeared. This process has been attributed to the decomposition of MoS_x_ into Mo nanoparticles embedded in a Li_2_S matrix through the conversion process^42^,^43^. Upon the anodic scan, the oxidation peak at ~1.5 V can be in part attributed to the oxidation of Mo to MoS_2_ followed by a anodic peak at 2.3 V associated with the oxidation of Li_2_S into S^42^,^43^,^45^. In addition, lithium extraction from Li_n_MoS_x_ could contribute to these anodic processes depending on the stoichiometry of the LinMoS_x_^39^. During the 2^nd^ CV scan, a pair of reduction peak at ~1.3 V and ~1.80 V together with two corresponding oxidation peaks at ~ 1.5 and 2.3 V for the MoS_x_/MWNTs composite became distinct. The reduction peak at ~1.3 V can be related to the intercalation of Li^+^ into the MoS_x_ lattice While, the oxidation peaks at ~1.48 V and 2.28 V correspond to the extraction of Li^+^ from Li_n_MoS_x_ lattice and the oxidation of Li_2_S, respectively^40^.

**Figure 5:**
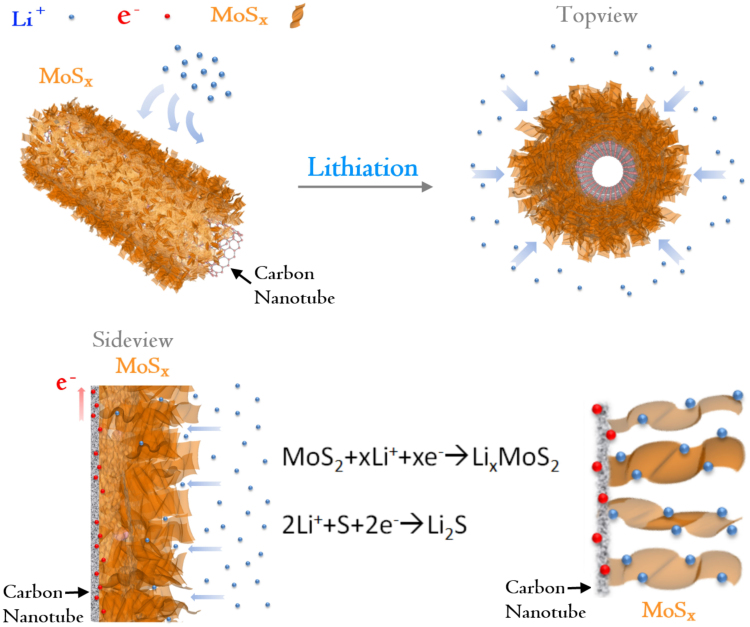
Schematic illustration of the diffusion of electron and Li. The Li ion can diffuse into the hierarchical MoS_X_/MWNTs nanocomposites easily from the open space between neighboring. Hierarchical structures enhance the contact area, shorten the Li ion diffusion length in the nanosheets, and ensure that Li and electron diffuse with little resistance.

Figure 4 (C) shows the cycling stability of the MoS_x_/MWNTs electrode compared to the pristine MWNTs. The specific capacity of the MoS_x_/MWNTs composite with a Mo/C molar ratio of 1:1 is above 1000 mAh/g which is more than 4 times larger than the pristine MWNTs electrodes under current density of 50 mA/g. The specific capacities of MoS_x_/MWNTs composites with various Mo/C molar ratios are shown in supporting information Figure S2. Figure 4 (D) shows the rate capability of the MoS_x_/MWNTs composites at various current densities. The electrode shows the 10^th^-cycle discharge capacities of 1119, 904, 659, 358 and 197 mAhg^−1^ at current densities of 50, 200, 500, 1000 and 2000 mAg^−1^, respectively. Even at a very high current density of 1000 mAg^−1^, the composite electrode can still deliver a capacity of 358 mAhg^−1^, which is comparable with the theoretical capacity of graphite (372 mAh g^−1^). Furthermore, after the current density returns from 2000 mAg^−1^ to 50 mAg^−1^, the specific capacity of MoS_x_/MWNTs electrode can recover to 1087 mAhg^−1^ and remain 1098 mAhg^−1^ after 10 cycles. Our MoS_x_/CNTs have shown a remarkably high reversible specific capacity (i.e., > 1000 mAh/g) at the current density of 50 mA g^−1^, which is much larger than the “theoretical” capacity value of MoS_2_ (670 mAh/g assuming 4 lithium ions per MoS_2_) and CNTs along. We note that specific capacity of MoS_2_ higher than 670 mAh/g is well-documented in the literature^45^,^48^,^15^. It was shown that MoS_2_ can take up to 8 lithium ions with major capacity between 0.01 to 1.0 V vs. Li/Li^+^ 15, which corresponds to a theoretical capacity up to 1334 mAh/g. It is believed that the lithium ions can be stored in different defect sites of the MoS_2_ depending on the morphology of the material^15^. In addition, Kartick et al. reported that MoS_2_/CNT composites prepared by dry grinding method can achieve a reversible storage capacity around 1000 mAh/g^49^ and X. Cao et al. reported that the MoS_2_ layers grown on CVD-G has a reversible capacity above 1000 mAh/g^50^. We believe that the high capacity observed in our study is associated with the unique material structure and defect distribution of MoS_x_/CNT. It worth mentioning that the MoS_x_/MWNTs composites had better rate performance compared to the reported single-layer MoS_2_-graphene composites^40^ and much improved cycling stability than the MoS_2_ electrodes^27^,^40^. As demonstrated by the schematical illustration image in Figure 5, the high rate capability can be attributed to the unique hierarchical nanoarchitecture of MoS_x_/MWNTs which provide structural stability and transport advantages for both electrons and lithium ions. The Li^+^ ion from the surrounding of MoS_x_/MWNTs have sufficient contact with the Li accommodate layers, and the exposed MoS_x_ edges provides abundant intercalations tunnels. The MWNTs provide fast electronic conduction channels and ensure the individual high specific MoS_x_ layerelectrically connected during charge/discharge cycles, meanwhile the Li^+^ are accommodated in the metal sulfide layers.

In conclusion, the outstanding performance of hierarchical composites based anode material is attributable to the unique synergy at the nanoscale between 1D CNT and Li^+^ hosting 2D nanoseets. The CNTs provide high conductance channels and ensure the individual high specific MoS_x _layerelectrically connected during charge/discharge cycles, meanwhile the Li^+^ are accommodated in the metal sulfide layers. Moreover, the designed hierarchical structure with maximized surface and increased layer distance of S–Mo–S have resulted in less strain and smaller intercalation barrier of Li ions, which maintain the high lithium storage in reversible capacities, stable cycling lifetime, and excellent rate performances. Other promising applications are also anticipated to arise that take advantage of the abundant active MoS_x_ edges as catalysts^51^,^52^,^53^,^54^.

## Methods

### Preparation of MoS_x_/MWNTs nanocomposite

The multi-walled carbon nanotubes (MWNTs), L-MWNTs-60100, were purchased from Shen-zhen Nanotech Port Co., Ltd, Shenzhen, China. The (NH4)_2_MoS_4_ powder and N,N-dimethylformamide (DMF) were purchased from Sigma-Aldrich. All chemicals and raw materials were directly used without further purification. The MWNTs/MoS_2_ hybrid was prepared by a solvent thermal process. In a typical experiment, 220 mg (NH4)_2_MoS_4_ powder (Sigma-Aldrich) and 100 mg MWNTs were mixed and dispersed into 30 ml of N,N-dimethylformamide (DMF) in a 40 ml Teflon autoclave. After that, the solution was sonicated at room temperature for approximately 10 mins until homogeneous solution was achieved. Then the autoclave was sealed tightly and heated at 200°C for 10 hours under autogenous pressure without intentional control of ramping and cooling rate. After cooled down to room temperature, the product was extracted by centrifugation at 10,000 rpm for 5 min. To remove the unreacted molecules and most of the DMF residuals the product was dispersed in DI water and recollected by centrifugation, this washing step was repeated for at least 5 times, the final products was MWNTs/MoS_x_ nano composite.

### Materials characterization

X-ray photoelectron spectroscopy (XPS) analysis was performed on a KRATOS AXIS ULTRA-DLD spectrometer with a monochromatic Al K_α1_ radiation (*hv* = 1486.6 eV). The morphologies and microstructures of the products were characterized by transmission electron microscopy (TEM) and high resolution TEM (HRTEM) on a JEM 2100F microscope. The Raman spectra were obtained by using WITec CRM 200 confocal Raman microscopy system with a laser wavelength of 488 nm and spot size of 0.5 μm. To calibrate the wavenumber, the Si peak at 520 cm^−1^ was used as a reference.

### Electrochemical measurements

The electrochemical performance of MWNTs/MoS_x_ nanocomposites electrode was measured with a half-cell lithium ion battery (LIBs) configuration. The 2032 coin-type cells were assembled in an argon-filled glove-box with both of the moisture and oxygen level less than 1 ppm. The working electrode material slurry were prepared by mixing MWNTs/MoS_x_, carbon black and poly(vinyldifluoride) (PVDF) at a weight ratio of 80:10:10, several drops of N-methylpyrrolidone (NMP) solvent was added into the mixture to prepare the active materials slurry. The resulting slurry was then uniformly pasted onto Ni foam, with mass loading of 4 ~ 6 mg. Lithium sheet was used as anodes and 1 M LiPF_6_ in a 1/1 (volume ratio) mixture of ethylene carbonate (EC)/dimethyl carbonate (DMC) as electrolyte. Cegard® 2400 was used as the separator of the battery. The cells were tested on a NEWARE multi-channel battery test system with galvanostatic charge and discharge in the voltage range between 0.01 and 3.0 V vs. Li/Li^+^ at various current density at room temperature. The cyclic Voltammetry (CV) and electrochemical impedance spectroscopy (EIS) were tested on an electrochemical workstation (VMP3, Bio-Logic).

## Author Contributions

Y.S. and H.Y.Y. conceived the project. Y.S., Y.W. and H.Y.Y. designed and carried out research, analyzed data. Y.S. and H.Y.Y. wrote the paper. A.Y.S.T., J.I.W. and C.L.H. contributed in material characterization and discussion. Y.C.L. and L.J.L. provide scientific advice. All authors contributed to the writing and editing.

## Supplementary Material

Supplementary Information Supporting Information
