# Supplementary material for: Self-assembly of hierarchical MoSx/CNT nanocomposites (2<x<3): towards high performance anode materials for lithium ion batteries
Source: Sci Rep. 2013 Jul 9;3:2169. doi: 10.1038/srep02169 (PMC3705413; doi:10.1038/srep02169)
Supplement: Supplementary Information — Supporting Information [file srep02169-s1.doc]

Subject areas

**BATTERIES, NANOSCALE MATERIALS, SYNTHESIS AND PROCESSING**

Correspondence and requests for materials should be addressed to H.Y. Yang ([yanghuiying@sutd.edu.sg](mailto:yanghuiying@sutd.edu.sg))

**Self-assembly of hierarchical MoS_x_/CNT nanocomposites (2<x<3): towards high performance anode materials for lithium ion batteries**

Yumeng Shi,^1^ Ye Wang,^1^ Wong Jen It,^1^ Alex Tan Yuan Sheng,^1^ Chang-Lung Hsu,^2,3^ Lain-Jong Li,^2,4^ Yi-Chun Lu,^5^ & Hui Ying Yang,^1, *^

1. Pillar of Engineering Product Development, Singapore University of Technology and Design, Singapore 138682, Singapore
2. Institute of Atomic and Molecular Sciences Academia Sinica, Taipei 10617, Taiwan
3. Department of Materials Science & Engineering, National Chiao Tung University, HsinChu 300, Taiwan
4. Department of Physics Natonal Tsing Hua University, HsinChu 300, Taiwan.
5. Department of Mechanical and Automation Engineering, The Chinese University of Hong Kong, People’s republic of China

**
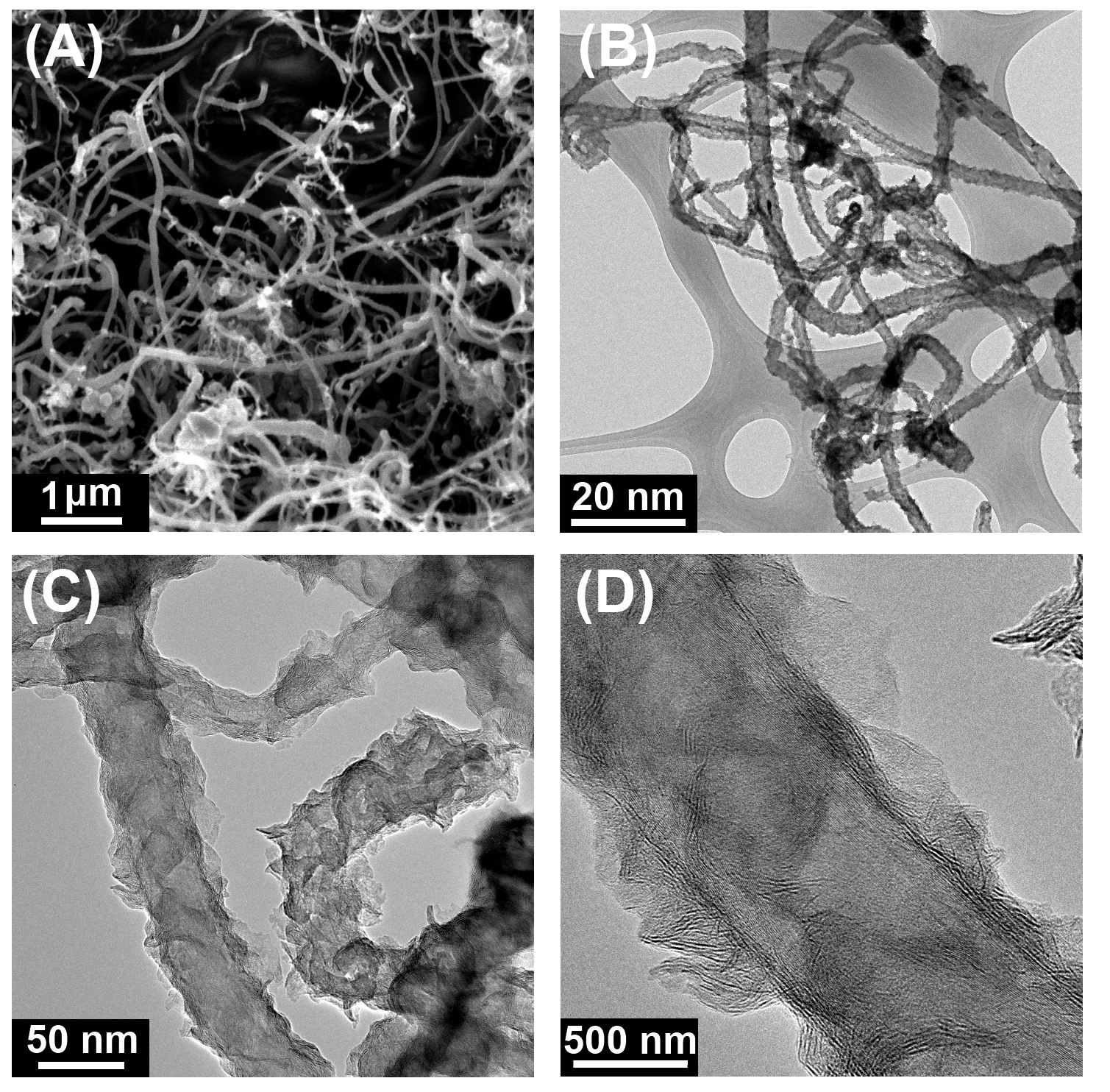
**

**Figure S1** (A) SEM image of Pristine MWNTs; (B), (C) and (D) show the TEM images of as prepared MoS_x_/MWNTs nanocomposites under various magnifications.

Figure S1. (A) shows the scanning electron microscopy (SEM) of pristine MWNTs used in the experiments. The multiwalled carbon nanotubes used in this work have an outer diameter ranging from 20 to 60 nm with a length of 5 to 15 μm. Figure S1. (B), (C) and (D) show the typical transmission electron microscopy (TEM) and high-resolution TEM (HRTEM) images of the synthesized MoS_x_/MWNTs composite at different magnification. Different from the previous report for MoS_2_ sheath/CNT-core nanoarchitecture, [^1^](#_ENREF_1) the MoS_x_ layers are not confined to the MWNTs surface, but extend the layered structure out of the cylindrical tubules.


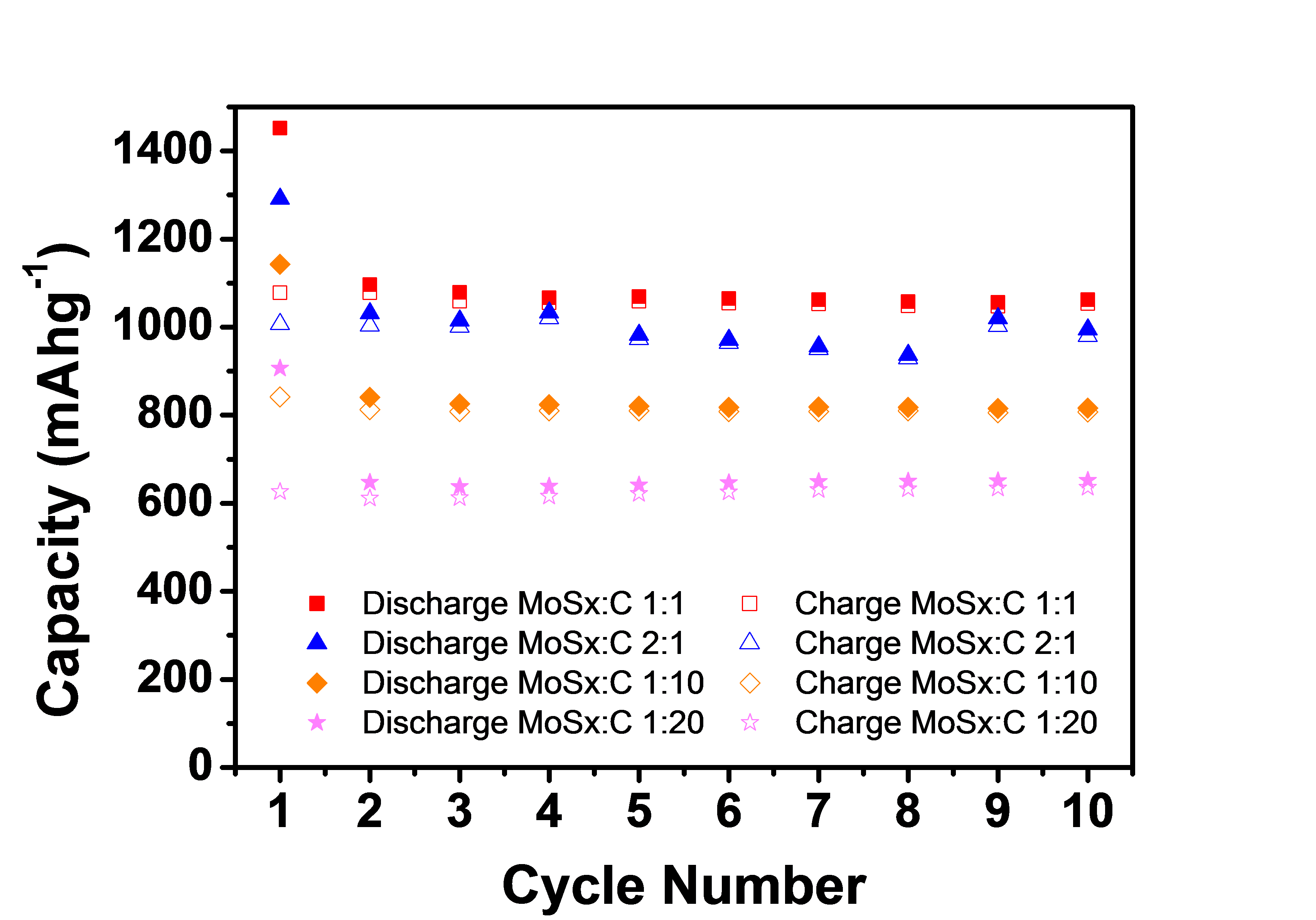


**Figure S2** Specific Capacity of MoS_x_/MWNTs with various MoS_x_/Carbon ratios. The battery were charged and discharged at 50 mA g^-1^. The capacities are calculated by the total weight of MoS_x_/MWNTs composites.


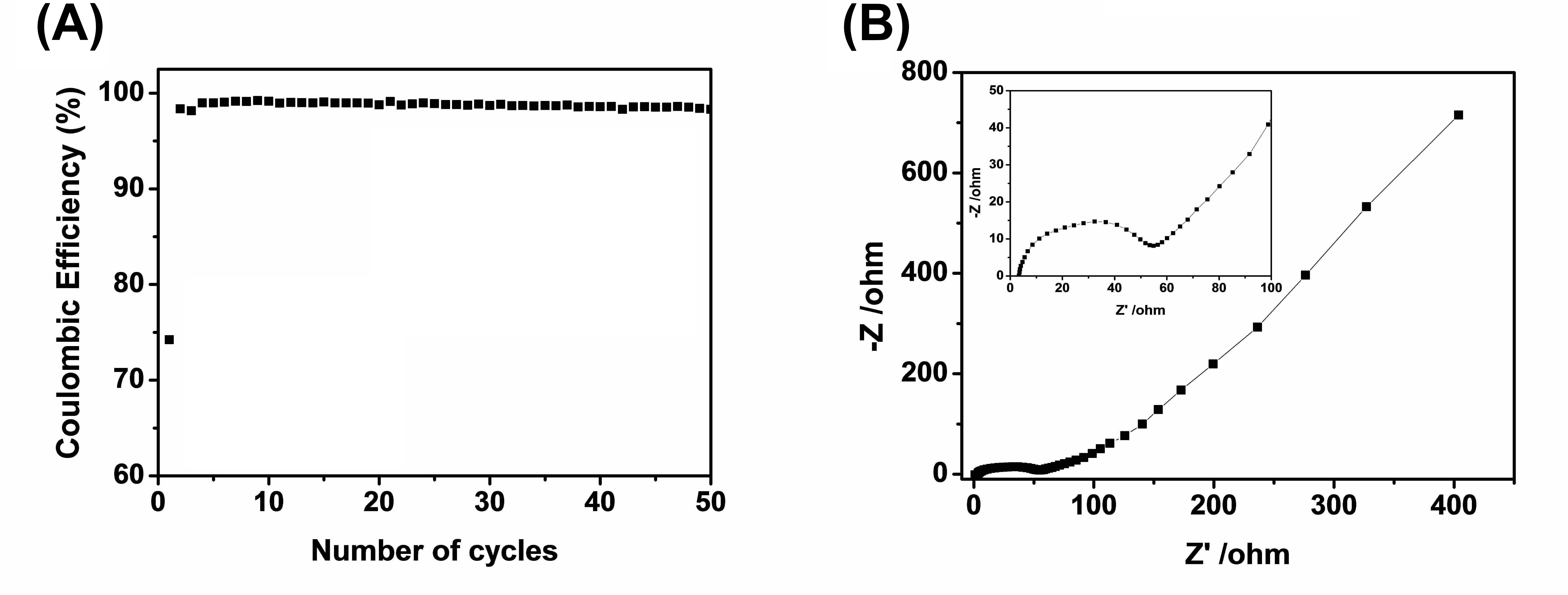


**Figure S3** (A) Coulombic efficiency of LIBs with MoS_x_/MWNTs anode electrode for the first 50 cycles; (B) Nyquist plots of MoS_x_/MWNTs electrodes after 10 discharge/charge cycles. Inset shows the magnified Nyquist plots of the high-frequency range of MoS_x_/MWTNs composite electrode.

Figure S3. (A) displays the Coulombic efficiency of the LIBs based on MoS_x_/MWNTs composites. The Coulombic efficiency (CE) refers to the ratio of the charge delivered during discharge to the amount stored during charging. The CE value fast increased to 98.3% for the second cycle and maintained above 98% for the rest cycling tests. To evaluate the electrical and ion transfer resistance between MoS_x_/MWNTs with Li ions, the superior Li storage properties of the MoS_x_/MWNTs composite electrode is tested by the electrochemical impedance spectra (EIS) measurements on the samples after 10 discharge/charge cycles. Figure S3. (B) shows the MoS_x_/MWNTs composite electrode with a small radius of semi-circle in the Nyquist plots. This suggests a low contact and charge-transfer resistances for the MoS_x_/MWNTs composite electrode.


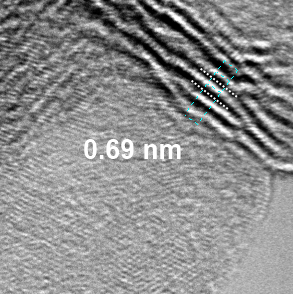


**Figure S4** HRTEM images of a free standing monolayer MoS_x_ on the side wall of the nanocomposite.

Figure S4 shows the layer distance measurement from the TEM. The layer distance of MoS_x_ is around 0.69 nm, which is a bit larger than the layer distance of MoS_2_ (0.63 nm) . The S-Mo-S forms chemical bonds from the XPS analysis. The layered distance depends on the synthesis conditions, for this composite it can be seen as S riched MoS_2_. The additional S may cause the increase layer to layer distance. CNT may not change the layer distance, but provide a growth template for S-Mo-S.

1 Wang, Q. & Li, J. Facilitated Lithium Storage in MoS2 Overlayers Supported on Coaxial Carbon Nanotubes*. The Journal of Physical Chemistry* **C 1**11, 1675-1682, doi:10.1021/jp066655p (2007).
